# Hemodynamic Functionality of Transfused Red Blood Cells in the Microcirculation of Blood Recipients

**DOI:** 10.3389/fphys.2018.00041

**Published:** 2018-01-30

**Authors:** Gregory Barshtein, Dan Arbell, Saul Yedgar

**Affiliations:** ^1^Department of Biochemistry, Faculty of Medicine, Hebrew University, Jerusalem, Israel; ^2^Department of Pediatric Surgery, Hadassah University Hospital, Jerusalem, Israel

**Keywords:** microcirculation, red blood cells, blood transfusion, RBC deformability, RBC adhesion, RBC hemodynamic functionality

## Abstract

The primary goal of red blood cell (RBC) transfusion is to supply oxygen to tissues and organs. However, due to a growing number of studies that have reported negative transfusion outcomes, including reduced blood perfusion, there is rising concern about the risks in blood transfusion. RBC are characterized by unique flow-affecting properties, specifically adherence to blood vessel wall endothelium, cell deformability, and self-aggregability, which define their hemodynamic functionality (HF), namely their potential to affect blood circulation. The role of the HF of RBC in blood circulation, particularly the microcirculation, has been documented in numerous studies with animal models. These studies indicate that the HF of transfused RBC (TRBC) plays an important role in the transfusion outcome. However, studies with animal models must be interpreted with reservations, as animal physiology may not reflect human physiology. To test this concept in humans, we have directly examined the effect of the HF of TRBC, as expressed by their deformability and adherence to vascular endothelium, on the transfusion-induced effect on the skin blood flow and hemoglobin increment in β-thalassemia major patients. The results demonstrated, for the first time in humans, that the TRBC HF is a potent effector of the transfusion outcome, expressed by the transfusion-induced increase in the recipients' hemoglobin level, and the change in the skin blood flow, indicating a link between the microcirculation and the survival of TRBC in the recipients' vascular system. The implication of these findings for blood transfusion practice and to vascular function in blood recipients is discussed.

## Introduction

Blood transfusion has long been considered a routine life-saving therapy that has revolutionized medicine (Diamond, 1980). Donated blood units, routinely stored as packed red blood cells (PRBC) are routinely stored for up to 35 or 42 days, depending on the preservation solution (Hess, 2006). However, in recent years, there has been a growing concern about the efficacy and safety of the transfusion of allogeneic stored blood (Glynn, 2008; Hillyer et al., 2008; Redlin et al., 2014), as many studies have shown that PRBC transfusion caused damage rather than benefit to recipients. This included prolonged mechanical ventilation, renal failure and sepsis, with increased hospitalization and mortality in transfusion recipients (Sherk et al., 2000; Ho et al., 2003; Gong et al., 2005; Aronson et al., 2008; Glynn, 2008; Hillyer et al., 2008; Koch et al., 2008; Leal-Noval et al., 2008; Marin et al., 2013; Almac et al., 2014; Zimring, 2015). In particular, studies with patients suffering from trauma (Weinberg et al., 2012), sepsis (Sakr et al., 2007; Damiani et al., 2015), or thalassemia (Vasileiadis et al., 2013), as well as patients in the medical-surgical intensive care (Creteur et al., 2009), have reported that PRBC transfusion reduced blood perfusion (Sakr et al., 2007; Weinberg et al., 2012), oxygen saturation (Creteur et al., 2009; Vasileiadis et al., 2013), oxygen delivery, and induced tissue hypoxia (Hayes et al., 1994).

Some studies have attributed that to storage-induced lesion to PRBC, showing better outcome with fresher vs. long-stored PRBC (Tinmouth and Chin-Yee, 2001; Gonzalez et al., 2007; Kor et al., 2009; Hess, 2010; D'Alessandro and Zolla, 2013; Damiani et al., 2015; Kim et al., 2015; Obrador et al., 2015; Antonelou and Seghatchian, 2016; Parviz et al., 2016).

However, other studies have not found relation between storage duration and negative transfusion outcome, showing no clinical benefit to using fresher over long-stored RBC (Remy et al., 2016; Shah et al., 2016). The role of storage duration on transfusion outcome is thus still a matter of debate (Lelubre and Vincent, 2013; Brunskill et al., 2015).

Blood donations are routinely tested, on day of donation, for immune compatibility and infectious agents, and are supplied by the first-in-first-out (FIFO) criterion. However, the functionality of transfused RBC, namely their ability to affect the transfusion outcome is ignored. RBC transfusion is aimed at increasing the recipients' hemoglobin and tissue oxygenation. However, RBC have unique flow-affecting properties, which play a key role in blood circulation, and define their hemodynamic functionality, namely their capacity to affect the recipients' blood circulation. The present review focuses on the role of transfused RBC FP on the recipients' blood circulation.

## RBC flow-affecting properties (FP) in blood circulation

RBC FP refer mainly to the cells' deformability, potential adherence to blood vessel all endothelium, and self-aggregability (Shiga et al., 1990; Barshtein et al., 2007; Simmonds et al., 2013).

**RBC deformability** is the cells' ability to adapt their shape to enable their passage through microvessels, especially the capillaries, which are narrower than the RBC. Reduced deformability (**increased rigidity**) hinders blood perfusion and impairs oxygen delivery in peripheral tissues (Parthasarathi and Lipowsky, 1999; Sakr et al., 2007; Matot et al., 2013). Rigid RBC can attenuate perfusion in peripheral tissues and directly block microvessels, capillaries in particular (Mchedlishvili, 1998; Cabrales, 2007). It has been shown that exchange transfusion of rigid, aldehyde-fixated RBC reduced the flow rate in swine (Pantely et al., 1988), and the functional capillary density in hamsters (Cabrales, 2007). RBC deformability is also a major determinant of their passage through the splenic vasculature; reduced deformability hinders the cells passage and increases splenic RBC sequestration and destruction (Warkentin et al., 1990; Mohandas and Chasis, 1993; An and Mohandas, 2008; Huang et al., 2014).

**RBC adherence** to endothelial cells (EC) of the blood vessel walls (“adherence”) is normally insignificant, but it is abnormally enhanced in many disease states. RBC/EC adhesion decreases blood flow and increases the residence time of RBC in the microcirculation (Yedgar et al., 2008). Enhanced RBC/EC adhesion contributes to microcirculatory disorders observed in diverse pathologies, particularly those associated with oxidative stress (OS). In particular, it has been suggested that micro-vessel occlusion observed in sickle cell disease and malaria, especially cerebral malaria, is due to the adherence of sickle/malaria-infected RBC to EC of the micro-vessel wall (Yedgar et al., 2008). RBC/EC adherence is thus considered a potent catalyst of micro-vessel occlusion (Hebbel et al., 1981; Kaul and Nagel, 1993; Kaul et al., 1998, 2008; Hebbel, 2000).

**RBC aggregability** refers to the cells' ability to form multi-cellular aggregates, normally in a rouleau shape, in the presence of plasma proteins, especially fibrinogen, or other macromolecules (Skalak et al., 1981; Barshtein et al., 2007). Under normal conditions, the flow-induced shear stress is sufficient to modulate the aggregation as physiologically required to enable adequate blood flow in the diverse blood vessels. However, in pathological states, mainly those with low-flow or RBC abnormalities, aggregates that are larger and stronger-than-normal are formed, and higher shear stress is required for their disaggregation (Chen et al., 1995; Ami et al., 2001). Elevated RBC aggregation has been shown to be associated with cardio-vascular diseases (Mohandas and Chasis, 1993; Barshtein et al., 2007), and found to be correlated well with inflammatory indices of patients with unstable angina, myocardial infarct and sepsis (Ami et al., 2001). Increased RBC aggregation elevates blood viscosity and is associated with the formation of an RBC-free layer at the wall of large blood vessels. Accordingly, some studies suggested that the increased viscosity elevates vascular resistance. However, other studies suggested that RBC aggregation facilitates blood flow due to the formation of a cell-free layer at the vessel wall. In addition the viscosity-elevated shear stress leads to the production of the vasodilator nitric oxide (Kim et al., 2006, 2009; Namgung et al., 2011; Cho et al., 2015; Katanov et al., 2015; Ng et al., 2016). These led to disparate views as to the role of RBC aggregation in circulatory functions and disorders.

It is well known that pathological conditions or experimental treatments of RBC usually affect multiple properties, whereas the studies of RBC FP have generally focused on one property at a time, thereby leaving the derivation of the specific, differential effect of individual FP unclear.

This question was specifically addressed in a study, in which the adherence of human RBC was differentially elevated by treatment with H_2_0_2_ concentration that increased the adherence without affecting the deformability. The perfusion of these RBC into rat mesocecum, in a medium which did not induced aggregation (free of macromolecules) induced a considerable elevation of vascular resistance in rat mesocecum (Kaul et al., 2008). In another study (Matot et al., 2008), rat blood was stored for 7 days, during which its RBC deformability was markedly decreased, while their adherence and aggregation were insignificant, which is typical of rat RBC (Schlager et al., 2010). The perfusion of this blood to rats reduced the liver oxygenation, which led to liver necrosis. These studies (Kaul et al., 2008; Matot et al., 2008) thus provide direct evidence for the independent contribution of RBC/EC adherence and deformability to circulatory disorders.

Notably, RBC aggregates, even with the high aggregability observed in pathological conditions, can be disaggregated by a relatively low shear stress, such as 3–4 dynes/cm^2^, while a RBC with increased adherence or reduced deformability might remain adherent or rigid at a shear stress of 30–40 dynes/cm^2^ (Yedgar et al., 2008). It thus seems that the potency of RBC aggregation to induce vascular occlusion is much less significant.

## Transfusion of PRBC and recipients' microcirculation

The effect of PRBC transfusion on the recipients' blood circulation has been investigated in numerous studies, which together presented inconclusive, even opposing results.

Nielsen et al. (2017) reviewed 17 studies to examine whether or not PRBC transfusion improves tissue oxygenation and/or the microcirculation in critically ill patients. They concluded that the heterogeneity of study designs, methodologies and study populations did not enable an appropriate meta-analysis. Yet, in the majority of cases, RBC transfusion failed to result in significant improvement in either tissue oxygenation or microcirculatory flow in ICU patients.

A number of studies have made attempts to answer the question whether the microcirculatory response to the transfusion is sensitive to the storage duration of the transfused PRBC (Walsh et al., 2004; Bennett-Guerrero et al., 2009; Kiraly et al., 2009; Weinberg et al., 2013; Yürük et al., 2013; Stowell et al., 2017). While some studies reported that long-stored units impaired circulatory functions, such as gastric mucosal oxygenation status (Marik and Sibbald, 1993), and perfused capillary vascular density (Weinberg et al., 2013), others have not observed a significant correlation between the PRBC storage duration and the transfusion-induced change in the recipients' circulatory functions (Walsh et al., 2004; Stowell et al., 2017).

## Role of recipients' pre-transfusion conditions in transfusion-induced change in circulatory functions

On these grounds, of particular interest are the studies that pointed to the pre-transfusion conditions of the patients as an important factor in the transfusion-induced change in the recipients' circulation. These studies (Casutt et al., 1999; Sakr et al., 2007; Creteur et al., 2009; Sadaka et al., 2011; Weinberg et al., 2012) have suggested that patients who had lowered tissue oxygenation or microcirculatory flow indices prior to transfusion, have benefitted by the transfusion, showing a significant improvement in these indices. Conversely, patients who had normal values of these indices showed either no improvement or a decline after transfusion.

This is further supported by our recent study, showing that the transfusion-induced change in the recipients' skin blood flow (ΔSBF) was inversely related to the recipients' SBF before transfusion (SBF_B_). ΔSBF decreased, and was even negative, with increasing SBF_B_ (Barshtein et al., 2016). This implies that patients with the most severe tissue oxygenation or microcirculatory derangements (prior to transfusion) benefit the most from the transfusion.

This phenomenon also provides partial explanation for the discrepancy between the above studies showing opposing effects of PRBC transfusion on the recipients' blood circulation (Friedlander et al., 1998; Sakr et al., 2007).

RBC deformability had been shown to decrease in critically ill patients (Friedlander et al., 1998). As noted above, RBCs with decreased deformability are assumed to hinder the passage through the microvessels. However, Friedlander et al. found that the RBC deformability of transfused septic patients was elevated by the PRBC transfusion, and suggested that this improvement is due to the replacement of previously rigidified cells with newer, more functional RBCs (Friedlander et al., 1998). Hence, transfusion may be deleterious in patients with adequate RBC deformability, but may have positive outcome in patients with reduced RBC deformability.

The mechanism of this phenomenon is not fully understood, but we can speculate that for patients with relatively rigid RBC, such as those with critical illness, e.g., sepsis (Baskurt et al., 1998; Condon et al., 2003; Donadello et al., 2015), the transfusion of PRBCs may enter to the bloodstream RBC with relatively higher deformability, resulting in the improvement of microcirculatory perfusion (Friedlander et al., 1998). This points to the effect of PRBC FP on circulatory functions in transfusion recipients.

## Role of transfused RBC flow-affecting properties (FP) in transfusion-induced change in recipients' blood circulation

As noted above, the experimental evidence in animal studies supports the hypothesis that FP of transfused RBC are important determinants of the transfusion outcome, especially the recipients' blood microcirculation. However, as already stated, “animal studies must be interpreted with a number of caveats, as animal biology may not reflect human biology” (Zimring, 2015).

To bridge this gap, we directly studied, for the first time in humans, the effect of the HF of TRBC, expressed by their deformability and adherence, on the immediate transfusion outcome. This was measured by the transfusion-induced change in the recipients' skin blood flow, ΔSBF, determined by the difference in SBF before (SBF_B_) and after transfusion. In this study we employed β-thalassemia major (TM) patients, who are treated with long-life, frequent blood transfusions (every 2–4 weeks). It was clearly found that ΔSBF increased with increasing deformability of the TRBC (Barshtein et al., 2016). This was further supported by the data of the individual patients who received four consecutive transfusions over a period of 8–10 weeks. For each of the nine patients, ΔSBF increased with increasing TRBC deformability (Barshtein et al., 2016). In some cases, when the TRBC deformability was low and SBF_B_ was relatively high, the transfusion reduced the recipients' SBF (ΔSBF < 0) (Barshtein et al., 2016).

In another study (unpublished), we have found that ΔSBF depended on the difference in deformability and adherence between the TRBC and the recipients RBC; when the PRBC adherence/rigidity were lower than those of the recipients' RBC, the recipients' blood flow was increased (ΔSBF > 0). Conversely, when the TRBC rigidity/adherence was higher, the recipients' blood flow was decreased (ΔSBF < 0). This corresponds to the suggestion of Friedlander et al. (1998) that PRBC transfusion to critically ill patients improved their blood circulation, as their RBC deformability is especially low.

Taken together, these findings demonstrate, for the first time in humans, the important role of the hemodynamic functionality of transfused RBC, as expressed by their FP, in transfusion outcome.

## Transfused RBC hemodynamic functionality and vascular function

Transfused RBC (TRBC) can modulate vascular function in differing and opposing ways via their effect on the plasma NO level. On one hand, TRBC may induce vasodilation in two ways: the release of ATP, which activates NO production in the blood vessel wall endothelial cells (EC) (Cao et al., 2009; Cortese-Krott and Kelm, 2014; Sikora et al., 2014), and the direct release of NO from the *S*-nitrosylated hemoglobin (SNO-Hb) (Bennett-Guerrero et al., 2007; Reynolds et al., 2007). On the other hand, a significant part of the TRBC are hemolyzed in the vascular system shortly after transfusion, and release free Hb, which is a scavenger of NO, thereby exerting vasoconstriction (Rusak et al., 2014; Damiani et al., 2015). Notably, the lysis of TRBCs has been shown to correlate with the fraction of the rigid, undeformable RBC in the TRBCs (Orbach et al., 2017), and transfusion of rigid PRBC causes elevation of cell-free Hb level in the bloodstream (Damiani et al., 2015).

Taken together, the HF of TRBC seems to play a complex role in modulating vascular function, which seems to depend on the ratio between the cells with normal HF and those with impaired HF. Yet, further investigation is required to elucidate this complex mechanism.

## Conclusion

The findings and considerations summarized above demonstrate the important role played by the HF of TRBC in the response of the recipients' vascular function. However, this is a complex response consisting of various effects of the deformability and adherence of the transfused RBC. These include direct effects on blood flow, primarily in the microcirculation, and their differing and opposing effects on vaso-modulation.

The present review presents direct evidence, in animal models and in humans, that the HF of transfused PRBC, as expressed primarily by their deformability and adherence to EC, is a potent effector of transfusion outcome. This strongly supports the need for considering the hemodynamic quality of transfused RBC in blood banking. The assessment of PRBC HF would introduce a powerful tool for reducing transfusion-related risks and improving transfusion therapy.

## Author contributions

GB, DA, and SY have been involved in the analysis and discussion of studies relating to the subject and in the writing of the review.

### Conflict of interest statement

The authors declare that the research was conducted in the absence of any commercial or financial relationships that could be construed as a potential conflict of interest.
